# The influence of Black Cohosh on hippocampal and hypothalamic gene expression profiles in ovariectomized rats and its potential to treat menopausal decrease in smell discrimination

**DOI:** 10.1007/s00404-024-07632-w

**Published:** 2024-07-24

**Authors:** Elena Pavicic, Katrin Rüst, Stefan Ehrentraut, Michael von Wolff, Petra Stute

**Affiliations:** 1 School of Medicine, University of Bern, Bern, Switzerland; 2 Department of Obstetrics and Gynaecology, Lucerne Cantonal Hospital, Luzern, Switzerland; 3 Arrows Biomedical Deutschland GmbH, Münster, Germany; 4 Department of Obstetrics and Gynaecology, University Clinic of Bern, Bern, Switzerland

**Keywords:** Cimicifuga racemose, Hippocampus, Hypothalamus, Olfactory system, Menopause

## Abstract

**Purpose:**

Menopause is associated with a decrease in smell discrimination ability. This study assessed the impact of black cohosh on hippocampal (HC) and hypothalamic (HT) gene expression profiles in rats, to understand, if herbal treatment has an impact on neurologic changes due to menopause and whether this could address a decrease in smell discrimination.

**Methods:**

HC and HT tissues from female Sprague Dawley rats (total *n* = 19) were analyzed at three different life stages: intact tissues of the HC (*n* = 4) and the HT (*n* = 4), oophorectomized tissues 3 months after oophorectomy (OVX) of the HC (*n* = 4) and the HT (*n* = 3), and tissues after treatment with an isopropanolic extract (iCR) from the rhizomes of black cohosh (60 mg/kg) for 3 months after OVX of the HC (*n* = 2) and the HT (*n* = 2).

**Main outcome measures:**

To reveal underlying biological processes a gene set enrichment analysis (GSEA) was performed.

**Results:**

The GSEA revealed gene ontology terms that were significantly enriched, including several genes associated with the olfactory system, indicating biological processes regulated by treatment with iCR. Six olfactory receptor genes were further analyzed by another GSEA, demonstrating the possibility of iCR treatment to compensate for oophorectomy-induced changes.

**Conclusion:**

Findings suggest that herbal treatment, such as iCR, has an esteeming impact on HC and HT genes that are changed through menopause. Further studies are needed to suggest black cohosh as a treatment option for decreased smell discrimination.

## What does this study add to the clinical work

**Table:** 

This study demonstrates that herbal treatment with cohosh (BC) signifcantly alters gene expression profiles in the hippocampus (HC) and hypothalamus (HT), particularly affecting the olfactory system. Clinically this suggests BC might help manage some sensory and physilogical changes during menopause, making it a potential option for future treatment.	

## Introduction

Our ability to qualitatively discriminate among different odors may no longer be essential for survival but certainly supports a high quality of life. Unfortunately, the sense of smell changes throughout different life stages. As people age, a decline in olfactory cells, nerve fibers, and mucus production, among other factors, leads to reduced olfactory capability in both healthy men and women [1]. Furthermore, postmenopausal women experience a notable increase in the threshold of perception due to declining estrogen levels. Diminishing estrogen levels result in reduced gray matter volume across various brain regions, including the supplementary motor area, inferior frontal gyrus, superior temporal gyrus, and olfactory cortex. This phenomenon elucidates several menopausal symptoms, notably the decline in olfactory capability [2–3].

Responsible for the detection of odorants are olfactory receptors (OLR). These are part of the large g-protein coupled receptors and are located within the nasal olfactory epithelium, reacting to odorant molecules thus leading to the perception of smell. The olfactory receptor gene family is the largest of the genome and other than in nasal epithelium, OLR genes can be found within many ectopic tissues, such as the brain, more precisely the hypothalamus (HT) and the hippocampus (HC) [4]. Both brain areas, HT and HC are involved in olfactory functions. The HT is part of the vital reaction of prey animals to predators, as their scent induces an instinctive fear response that includes behavioral changes, as well as an increase in blood stress hormones that mobilizes multiple body systems to escape impending danger [5]. Multiple olfactory cortical areas transmit signals to hypothalamic corticotropin-releasing hormone (CRH) neurons which control stress hormone levels [6]. Ensuring survival, olfactory perception in mice modulates food intake depending on the energy balance of the body through the neuropeptide orexin A, a hypothalamic neuropeptide. On the other hand, gonadotropin-releasing hormone (GnRH) released by the nervus terminals at the level of the olfactory epithelium, is able to reduce sensitivity to food odorants in the olfactory epithelium so that olfactory system is predominantly available for odorants involved in mating [7]. The HC, for its part, plays a role in olfactory perception in mice, as repeated food intake changes neuronal signaling in the HC suggesting that mice are able to learn food approach behavior [8]. Furthermore, olfactory dysfunction is reported to be a possible early symptom of Alzheimer’s disease, where the HC similarly plays an important role [9]. Overall, olfactory function is therefore a vital necessity working through interactions with both the HT, as well as the HC.

To compensate for the decrease in smell discrimination, hormone replacement therapy (HRT) has proven likely to be beneficial [10]. However, there is still a variety of different research outcomes whether HRT has an effect on cognitive and olfactory functions, while a recent study suggests a positive effect [11–12]. The different outcomes depend, for example, on the duration of treatment [13], on estrogen replacement therapy (ERT) only or combined HRT (estrogen and progestogen) [14] and also if the study involved women possessing the APOE-ε4 allele, which contains susceptible genes for Alzheimer’s disease. A brain region that is positively affected by long-term low-dose HRT is the HC in women expressing the ApoE-ε3/-ε4 genes, where HRT prevents from HC atrophy and is therefore also reducing the risk of developing Alzheimer’s disease [15–16]. Currently, HRT is mainly recommended to alleviate vasomotor symptoms, such as hot flushes caused by hypothalamic mechanisms. However, HRT is not recommended for maintenance of olfactory function after menopause [17]. Even though a recent comprehensive review reports HRT to be safe for women who use low-does HRT for short durations, younger women and women within 10 years of menopause [13], a history of heart attack, stroke or breast cancer—to mention only a few—would put women at high risk, when using HRT [18]. Thus, many menopausal women seek for alternative natural therapies to HRT.

Addressing this problem, there is growing interest in the use of black cohosh (BC, *Cimicifuga racemosa*) [10]. BC has been found to significantly reduce hot flushes possibly by increasing the number of c-fos protein—a marker of neuronal activity—and positive cell density within the HT nuclei [19–20]. BC also interacts with the hypothalamic–pituitary–adrenal axis thereby alleviating the acute stress responses in rats [21]. Furthermore, BC has been shown to display neuroprotective effects in rats and to modulate hippocampal local steroid metabolism in non-human primates [22–23]. A recent meta-analysis comprising 35 clinical studies showed that neurovegetative and psychological menopausal symptoms in women were effectively reduced through isopropanolic *Cimicifuga racemose* (iCR) extract when compared to placebo [24].

In the present study, we assessed gene expression profiles within the HC and HT of rats with or without BC treatment. Our hypothesis was that BC alters the hippocampal and hypothalamic gene expression profiles in oophorectomized rats compared to intact rats. A confirmation of this hypothesis would strengthen the use of black cohosh as a treatment for decreased smell discrimination due to neurologic changes during menopause.

## Methods and materials

### Animal characteristics

Hippocampal and hypothalamic tissue from Female Sprague Dawley rats (total *n* = 19), that were euthanized in the course of other experiments [25], were collected and then analyzed at three different points of time presenting intact, oophorectomized and hormonally treated tissues. Group 1 (PRAE) presents intact hippocampal (*n* = 4) and hypothalamic (*n* = 4) tissues from rats without oophorectomy (OVX). Group 2 (OVX) presents oophorectomized hippocampal (*n* = 4) and hypothalamic (*n* = 3) tissues from rats 3 months after OVX. Group 3 (OVX + iCR) presents hippocampal (*n* = 2) and hypothalamic (*n* = 2) tissues from rats after treatment with an isopropanolic extract from the rhizomes of Cimicifuga racemosa (black cohosh, 60 mg/kg) for 3 months after OVX.

### Gene expression microarray assays

Total RNA was extracted from frozen hippocampal and hypothalamic tissue samples using QIAzol reagent followed by purification using a miRNeasy Mini kit (Qiagen, Hilden, Germany), and quantified using a Nanodrop UV–VIS spectrophotometer (Implen GmbH, München, Germany). RNA intactness and quality were confirmed using an Agilent 2100 Bioanalyzer (Wilmington, DE). Only samples with an RNA integrity number (RIN) greater than 8.0 were used for hybridization. 100 ng of total RNA from each sample was labeled using the Low Input Quick Amp Labeling kit (Agilent Technologies Inc., Santa Clara, CA) following the manufacturer’s one-color microarray-based expression analysis protocol. RNA was then fragmented and hybridized to Gene Expression 4 × 44 K Rat Genome Arrays (Agilent Technologies Inc., Santa Clara, CA) for 17 h, prior to washing and scanning. Data were extracted from scanned images using the Agilent feature extraction software (Agilent Technologies Inc., Santa Clara, CA).

### Quantitative RT-PCR

RNA was extracted as described above. cDNA was subsequently synthesized from 3 µg RNA by random priming using the Superscript II Reverse Transcriptase (Invitrogen, Carlsbad, CA). Transcript levels for targets found to be significantly up- or down regulated were measured in quantitative real-time polymerase chain reaction (qRT-PCR) using the Sibir Hot Master Mix (BIORON GmbH, Ludwigshafen, Germany). Oligonucleotides were purchased from Biomers GmbH (Ulm, Germany). PCR was performed using the Light Cycler^®^ 480 Real-Time PCR System (Roche diagnostics, Risch, Switzerland). Samples were normalized to endogenous GAPDH and ACTB using rat-specific primers. Relative expression was determined using the ΔΔCt method calculated by the appropriate Light Cycler Software v1.5.

### Differential gene expression and statistical analysis

RNA was extracted from rat hippocampal and hypothalamic probes, from animals representing pre- or postmenopause. Differential gene expression analysis was performed setting contrasts between pre- and postmenopause (i), premenopause iCR treated (ii) and postmenopause iCR treated (iii) for hippocampus and hypothalamus, respectively. The cutoff was set as significantly (p-value < 0.05) up- or down-regulated genes that show a fold change (FC) of > 1.5 in gene expression. The R-based Bioconductor Linear Models for Microarray Data (LIMMA) package was used for statistical analysis [26]. The latest release of gene annotation data was purchased from the Bioconductor homepage (http://www.bioconductor.org). Gene ontology and pathway analyses were performed using the Broad Institute Gene Set Enrichment Analysis (GSEA) platform, which is a computational method that determines whether an a priori defined set of genes shows statistically significant, concordant differences between two biological states (e.g. phenotypes) [27].

## Results

### Global gene expression profiles

**Fig. 1:**
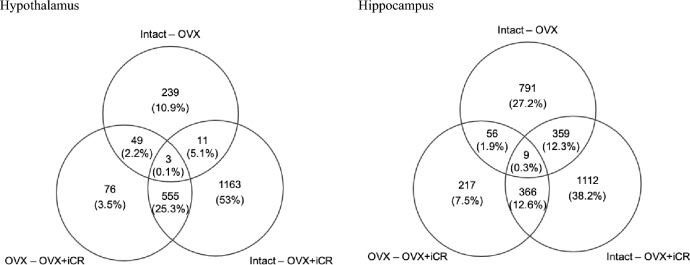
Commonly regulated genes comparing pre- to post menopause and iCR effect in Hypothalamus and Hippocampus Venn analysis showing commonly regulated genes in hypothalamic (HT) samples in and hippocampal (HC) samples in. Contrasts correspond to groups comparing (i) intact and OVX, (ii) intact and OVX + iCR and (iii) OVX and OVX + iCR for hippocampus and hypothalamus, respectively

In a total of 1957 genes in the hypothalamic and in 2119 genes in the hippocampal probes, treatment with iCR induced an up- or down-regulation of FC > 1.5 as shown in Fig. 1. In HT tissues 683 of 1957 genes were significantly up- or down-regulated by iCR when comparing OVX to OVX + iCR. In HC tissues the corresponding number was 648 of 2119 genes. To focus on the pharmacological effect of iCR in OVX samples we used an intersection analysis visualized in Venn diagrams (Fig. 1).

This analysis was used to select genes that are exclusively regulated by iCR without overlapping effects of pre- (intact) and postmenopause (oophorectomized). Thereby, we obtained 76 candidate genes in hypothalamic tissue and 217 candidate genes in hippocampal tissue, respectively. To reveal underlying biological processes, we performed a gene set enrichment analysis (GSEA) [28]. This analysis revealed pathways and gene ontology terms that were significantly enriched, indicating biological processes regulated by treatment with iCR as shown in Table 1 for gene expression in HC and Table 2 for gene expression in HT. Among these processes were several bone morphogenic or cartilage-related terms for the hippocampus probes, demonstrating the link between iCR and bone biology as reported before [29]. Furthermore, there was an enrichment of terms related to the immune system, reproductive behavior and responses to abiotic stimulus supporting a previous study in mice [30]. This gene expression profile revealed a clear response to the iCR treatment, further strengthened by the fact that also hypothalamic tissue showed an enrichment of genes associated with a response to inorganic substance, although through a different set of genes. In the next step, the question was addressed if iCR treatment-induced biological processes compensating for the pre- and postmenopausal effects. If this was the case, gene candidates should be found within the overlap between the group i (intact and ovx) and iii (ovx and ovx + iCR). Therefore, a GSEA was performed with 49 genes of the hypothalamus and 56 genes of the hippocampus, respectively (Fig. 1). Interestingly, most of these sets of genes are associated with the sensory system, specifically the olfactory system as shown in Table 3 for the HC and Table 4 for the HT.

**Table 1:** Gene expression microarray data in hippocampus

Cluster	Term	Count	% Enrichment	*p*-Value	Genes
1	GO:0060348 ~ bone development	5	2.59067358	0.026	ZCCHC2, MGP, COL1A1, SOX9, COL10A1
	GO:0060349 ~ bone morphogenesis	3	1.55440415	0.027	COL1A1, SOX9, COL10A1
	GO:0051216 ~ cartilage development	4	2.07253886	0.029	MGP, COL1A1, SOX9, COL10A1
	GO:0001501 ~ skeletal system development	7	3.62694301	0.033	ZCCHC2, COL3A1, MGP, COL1A1, SOX9, ALX3, COL10A1
2	GO:0019098 ~ reproductive behavior	3	1.55,440,415	0.015	DRD5, TH, TGM4
	GO:0007618 ~ mating	3	1.55,440,415	0.021	DRD5, TH, TGM4
3	IPR013162:CD80-like, immunoglobulin C2-set	4	2.07253886	0.005	BTNL8, KIRREL, BTN1A1, NPHS1
	GO:0002684 ~ positive regulation of immune system process	7	3.62694301	0.019	MASP2, ICOS, ITGA2, IL7R, SELE, TLR8, LAG3
	rno04672: Intestinal immune network for IgA production	3	1.55440415	0.059	CCR9, ICOS, CXCL12
4	GO:0009628 ~ response to abiotic stimulus	9	4.66321244	0.029	ZCCHC2, DRD5, COL3A1, MGP, ITGA2, CHEK2, COL1A1, SYNGAP1, CXCL12

Set of 217 genes regulated by iCR in hippocampus

**Table 2:** Gene Expression Microarray Data in Hypothalamus

Cluster	Term	Count	% Enrichment	*p*-value	Genes
1	GO:0010035 ~ response to inorganic substance	4	5,97014925	0.062	SLC25A13, NR3C1, FOSL1, ADAM9

Set of 76 genes, regulated by iCR in hypothalamus

**Table 3:** Gene expression microarray data in hippocampus

Cluster	Term	Count	% Enrichment	*p*-value	Genes
1	GO:0004984 ~ Olfactory receptor activity	9	19	0.002	OLR1063, OLR655, LOC690821, OLR780, OLR1200, OLR384, OLR1514, OLR841, OLR200
	GO:0009593 ~ detection of chemical stimulus	8	17	0.007	OLR1063, OLR655, OLR780, OLR1200, OLR384, OLR1514, OLR841, OLR200
2	GO:0050877 ~ neurological system process	10	21	0.005	OLR1063, COLQ, OLR655, OLR780, OLR1200, OLR384, OLR1514, OLR841, OLR200, TAS2R121

Set of 56 genes, regulated by iCR in hippocampus, overlapping to differential expression between pre- and post-menopause

**Table 4:** Gene Expression Microarray in Hypothalamus

Cluster	Term	Count	% Enrichment	*p*-value	Genes
1	GO:0004984 ~ Olfactory receptor activity	9	18	0.030	OLR84, LOC686683, OLR379, OLR823, OLR661, OLR1540, OLR156, OLR1521, OLR522
	GO:0009593 ~ detection of chemical stimulus	8	16	0.064	OLR84, OLR379, OLR823, OLR661, OLR1540, OLR156, OLR1521, OLR522
2	GO:0050877 ~ neurological system process	10	20	0.076	OLR84, HRH1, OLR379, OLR823, TACR2, OLR661, OLR1540, OLR156, OLR1521, OLR522

Set of 49 genes, regulated by iCR in hypothalamus, overlapping to differential expression between pre- and post-menopause

### Olfactory receptors

Looking at absolute data (not shown) and FC of up- or down-regulation of genes associated with the olfactory system, in some olfactory receptor (OLR) genes OVX induced a significant up- or down-regulation of hippocampal and hypothalamic genes that was compensated by iCR treatment, as documented in Tables 5 and 6. Interestingly, if OVX leads to a change in OLR gene expression, it almost exceptionally lead to an up-regulation and the following iCR treatment to a compensation, meaning a down-regulation. Also, the OLR genes that showed a reaction to OVX and iCR treatment differ in the HC and the HT. In the next step six of these OLR-genes (OLR379, OLR522, OLR655, OLR841, OLR661, OLR1063) were picked to be further validated by a quantitative real-time PCR (qRT-PCR) addressing the question, whether an iCR treatment would significantly and completely compensate OVX induced changes in these genes in the HT and HC.

**Table 5:** Fold changes up or down regulations of OLR-genes in Hippocampus

Gene	Intact – OVX	Intact – OVX + iCR	OVX – OVX + iCR
OLR84	2.94252	1.21937	−2.41314
OLR379	2.50644	1.4785	−1.69526
OLR823	4.04127	1.14747	−3.5219
OLR661	−2.8678	1.8085	5.18641
OLR1540	2.58238	1.221	−2.11498
OLR156	3.09302	1.25109	−2.47227
OLR1521	3.17143	1.30024	−2.43912
OLR522	2.07444	1.10047	−1.88505
OLR1063	1.3081	1.43127	1.09416
OLR655	1.01564	1.25382	1.23452
OLR780	−1.01442	1.18257	1.19962
OLR1200	1.09106	1.26118	1.15592
OLR384	1.00301	1.20911	1.20549
OLR1514	1.05776	1.2488	1.1806
OLR841	1.24981	1.67318	1.33875
OLR200	1.35285	1.25361	−1.07916

Up- and down regulation (fold change) of olfactory receptor genes by OVX + iCR

**Table 6:** Fold changes of up or down regulations of OLR-genes in Hypothalamus

Gene	Intact—OVX	Intact—OVX + iCR	OVX—OVX + iCR
OLR84	1.26195	1.18001	−1.06944
OLR379	1.27406	1.92163	1.50827
OLR823	1.3519	1.75897	1.30111
OLR661	2.85797	1.2186	−2.34529
OLR1540	1.09537	1.18173	1.07884
OLR156	1.12103	2.1536	1.92109
OLR1521	1.2533	1.21543	−1.03116
OLR522	1.66456	1.94379	1.16775
OLR1063	3.31585	1.27204	−2.60672
OLR655	2.06269	−1.03806	−2.14119
OLR780	2.26661	−1.06755	−2.41972
OLR1200	4.77291	1.03744	−4.60064
OLR384	2.9143	1.18632	−2.45659
OLR1514	2.03514	1.17995	−1.72477
OLR841	3.62671	1.00296	−3.61602
OLR200	2.483	−1.03652	−2.57368

Up- and down regulation (fold change) of olfactory receptor genes by OVX + iCR

Looking at the FC shown in Table 5, we assumed a nearly complete compensation of OVX-induced up-regulation by iCR treatment in OLR522 and OLR379 and a compensation of the OVX-induced down-regulation in OLR661. As for the HT, looking at the FC shown in Table 6, we assumed a compensation of OVX-induced up-regulation in OLR1063, OLR655 and OLR841.

**Fig. 2:**
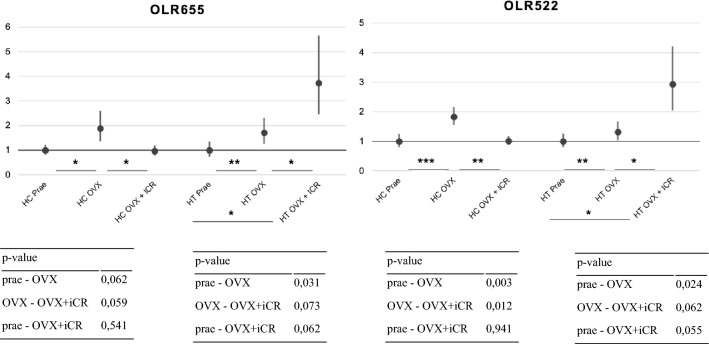
qRT-PCR showing the fold change and the corresponding p-value of OLR522 and OLR655 in the hippocampus and hypothalamus, respectively. * *p*-value < 0.1, **  *p*-value < 0.05, *** *p*-value < 0.01

**Fig. 3:**
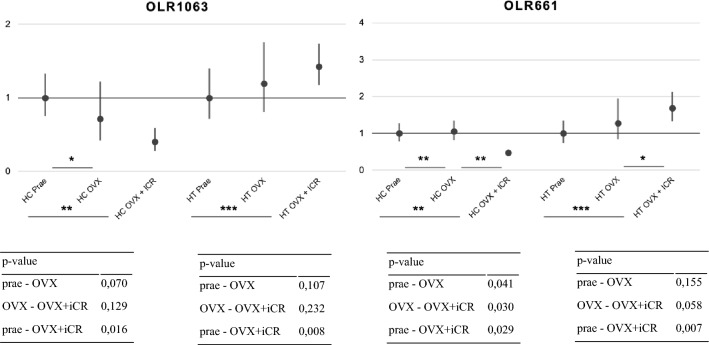
qRT-PCR showing the fold change and the corresponding p-value of OLR661 and OLR1063 and the *p*-value in the hippocampus and hypothalamus, respectively. * *p*-value < 0.1, ** *p*-value < 0.05, ***  *p*-value < 0.01

In hippocampal OLR522 the OVX induced an up-regulation by FC > 1.5 (p-value 0.003), as seen in Fig. 2. ICR treatment then induced a down-regulation by FC > 1.5 (p-value 0.012). The differential expression between intact and OVX + iCR is not statistically significant (p-value 0.941), thus proving a complete compensation of OVX-induced changes by the iCR treatment. Hypothalamic OLR522 did not show the same outcome as it is up-regulated by an OVX and further up-regulated after an additional iCR treatment, although it is not statistically significant (p-value 0.62), also seen in Fig. 2. The same OVX and iCR-induced changes can also be seen in hypothalamic OLR655, OLR661 and OLR1063. In hippocampal OLR655 the OVX-induced up-regulation is also reversed by the ICR treatment, but not statistically significant (p-value > 0.05). In hippocampal OLR379 and OLR841 an OVX induced an up-regulation (all p-values < 0.05), which the iCR treatment compensates by down-regulating the expression, but by a greater FC than the up-regulation, in terms of an overcompensation. In hippocampal OLR661 and OLR1063 an OVX induced no change or a down-regulation, with a further down regulation through iCR treatment (Fig. 3).

## Discussion

The present study investigated the impact of herbal treatment, with BC, on the olfactory system within the HC and the HT. Our data showed that treatment with BC has a significant impact on HT and HC gene expression profiles. A total of 1957 HT genes and 2119 HC genes were up- or down-regulated by 1.5 FC through an iCR treatment. A GSEA of these genes revealed that many of the influenced biological processes involved the sensory system, next to bone morphogenic, cartilage, immune system, reproductive behavior and responses to abiotic stimuli. Thus, proving that OVX and iCR treatment have a great influence on these pathways. Addressing the further question if iCR treatment is able to compensate for OVX-induced changes another GSEA was performed, revealing mostly olfactory receptor genes.

Six OLR genes that showed a reaction to the OVX and the iCR treatment, namely three HC genes and three HT genes, were further selected for a qRT-PCR to see if iCR treatment would completely compensate the OVX-induced up-regulation. Solely hippocampal OLR522 showed the expected results with a significant up-regulation by FC > 1.5 by OVX, followed by a down-regulation of FC > 1.5 through iCR treatment. HC OLR655 also showed the expected result as described, but only as a trend. And HC OLR379 showed a reaction to both the OVX and iCR treatment, but not by an FC > 1.5, and also only as a trend. None of the HT qRT-PCR validated OLR genes showed the expected result, as iCR further up-regulated the OVX-induced OLR gene expression. Overall, our data provides evidence that iCR treatment has an impact on many different biological pathways in both the HC and the HT.

The question remains of what impact a different expression of a single gene has on a whole pathway such as the olfactory function pathway. The GSEA was in fact explicitly invented to look at groups of gene expression profiles, rather than to look at single genes. Especially for the olfactory receptor gene family being the largest of the genome, we cannot tell if the differential expression of single genes would actually lead to a change in the olfactory function. The limitations of this study are therefore the small amount of gene expression profiles being analyzed by the GSEA. Also, looking at gene expression profiles does not reveal changes in the olfactory function in vivo.

**Fig. 4:**
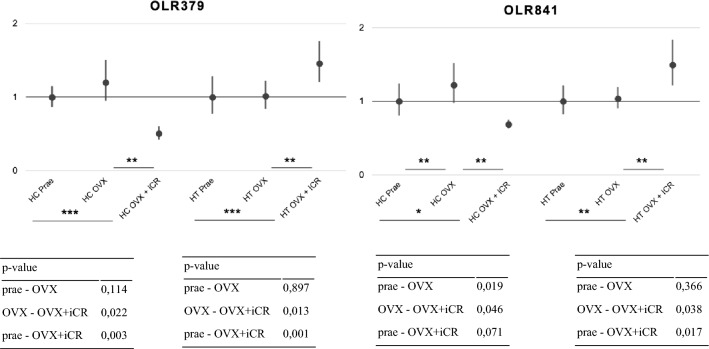
qRT-PCR showing the fold change and the corresponding p-value of OLR379 and OLR841 in the hippocampus and hypothalamus, respectively. * *p*-value < 0.1, ** *p*-value < 0.05, ***  *p*-value < 0.01

In order to draw more reliable practical conclusions, more research is needed. Future studies could, for example, involve an analysis of a greater number of gene expression profiles. Furthermore, randomized clinical trials would be of interest, since oophorectomy is not entirely identical to the processes occurring during menopause (Fig. 4).

## Conclusion

To conclude, our data showed an up-regulation of HC and HT olfactory receptor genes through an OVX. Crucially, we were able to corroborate our hypothesis, that herbal therapy with BC alters olfactory gene expression within the HC and the HT. Further studies would be needed to see if BC has an influence on the olfactory function in rats, since changes in single gene expression profiles may not lead to an altered OF. Thus, black cohosh cannot conclusively be recommended as a preventive treatment for postmenopausal olfactory functional changes. A major limitation of this study is the low sample size; the interpretation should be tentative.

## Data Availability

The authors confirm that the data supporting the findings of this study are available within the article and its supplementary materials.

## Abbreviations

BC: Black cohosh
CRH: Corticotropin releasing hormone
ERT: Estrogen replacement therapy
FC: Fold change
GnRH: Gonadotropin releasing hormone
GPCR: G-protein-coupled receptors
GSEA: Gene set enrichment analysis
HC: Hippocampus
HRT: Hormone replacement therapy
HT: Hypothalamus
iCR: Isopropanolic extract
OLR: Olfactory receptor
OVX: Oophorectomy
OVX + iCR: Treatment with iCR after OVX
PCR: Polymerase chain reaction
PRAE: Tissues of rats without OVX
qRT-PCR: Quantitative real-time PCR
